# Assessment of the correlation between various risk factors and orofacial cleft disorder spectrum: a retrospective case-control study

**DOI:** 10.1186/s40902-020-00270-7

**Published:** 2020-08-08

**Authors:** Behzad Cheshmi, Zahra Jafari, Mohammad Ali Naseri, Heidar Ali Davari

**Affiliations:** 1 Department of Oral and Maxillofacial Medicine, Faculty of Dentistry, Broujerd Branch, Islamic Azad University, Broujerd, Iran; 2 Department of Orthodontics and Dentofacial Orthopedics, Broujerd Branch, Islamic Azad University, Broujerd, Iran; 3 Craniofacial & Cleft Research Center, Isfahan University of Medical Sciences, Isfahan, Iran

**Keywords:** Cleft lip, Cleft palate, Orofacial cleft, Miscarriage, Abortion, Pregnancy

## Abstract

**Background:**

Orofacial clefts (OFCs) comprise a wide range of malformations, including cleft lip, cleft palate, and cleft lip with cleft palate, which can vary in terms of etiology, severity, and disease burden.

**Objective(s):**

This study aimed to evaluate the correlation between various risk factors and orofacial cleft disorder spectrum in newborns.

**Study design:**

A total of 323 cases and 400 controls were enrolled in this study and evaluated in terms of the maternal history of abortion or miscarriage, child’s sex, maternal and paternal age, maternal history of systemic disease, history of medication therapy during pregnancy, birth order, consanguineous marriage, and complications during pregnancy.

**Results:**

Analysis of the results suggested that consanguineous marriage, a maternal history of abortion/miscarriage, and complications during pregnancy could potentially increase the risk of OFCs in children (*P* < 0.05). However, the analyses revealed that the other variables could not potentially increase the risk of OFCs (*P* > 0.05).

**Conclusion(s):**

Multiple cofactors may simultaneously contribute to the formation of such abnormalities; therefore, a comprehensive, multidisciplinary care program is necessary to ensure a successful pregnancy period and the birth of a healthy newborn.

## Introduction

Orofacial clefts (OFCs) comprise a wide range of malformations, including cleft lip, cleft palate, and cleft lip with cleft palate, which can vary in terms of etiology, severity, and disease burden [1]. The etiology of OFCs is multifactorial, including various genetic (e.g., chromosomal abnormalities and syndromes) and environmental (e.g., medication use, dietary deficiencies, smoking, consumption of alcohol, obesity, exposure to toxins, high altitude, birth order, socioeconomic status, and parental age) factors [2–6]. Therefore, by understanding the multifactorial etiology of these abnormalities, we can explain the proposed approaches for the prevention and treatment of OFCs [7, 8]. Environmental factors, either alone or in combination with genetic factors involved in morphogenesis (e.g., TGF-α/TGF-β, BCL3, and MSX1), are commonly responsible for 60% of OFC abnormalities [4]. A wide range of pharmaceutical products, such as medications, which are generally used for the treatment of cancer, arthritis, and psoriasis, may also cause OFCs [9]. In one of the most comprehensive global studies of OFC prevalence, researchers found that 45,193 out of 30,665,615 people were diagnosed with one of these abnormalities, that is, 1.47 per 1000 live births worldwide [10]. Patients with a type of OFC often experience complications, such as cosmetic problems, reduced muscle function, feeding problems, ear infections, speech difficulties, dentoalveolar disorders (e.g., dental crowding and decay), and significant psychological effects on the child and parents [11, 12]. Currently, studies on individuals with OFCs mainly focus on the etiology of these abnormalities to reduce the incidence rate. Since the formation of OFCs is not often dependent on a single factor, and multiple cofactors may simultaneously contribute to such abnormalities, a one-dimensional study is unlikely to yield reliable results. Therefore, in this study, we aimed to evaluate the association between various factors and the birth of a child with OFCs.

## Materials and methods

This retrospective, case-control study was conducted at the Craniofacial and Cleft Research Center of Isfahan University of Medical Sciences, Isfahan, Iran, from 2014 to 2020. Data were collected by reviewing the medical records of mothers, fathers, and children, with or without a reported history of OFCs. The study procedures were verbally explained to the parents, and written consent was obtained. All procedures in this study were in accordance with the ethical standards of the local research committee, as well as the Declaration of Helsinki.

Mothers who received childbirth care at the hospital and delivered newborns with or without OFCs were included in this study. The exclusion criteria were as follows: (1) Patients without medical records, (2) patients with incomplete medical records, (3) mothers not receiving medical care at the hospital during 2014–2020 and also, (4) patients diagnosed with syndromic OFCs were excluded. The study variables included the maternal history of abortion or miscarriage, child’s sex, maternal and paternal age, maternal history of systemic disorders, history of medication therapy during pregnancy, birth order, consanguineous marriage, and complications during pregnancy.

The obtained data were stored in a database and analyzed using SPSS® Version 26.0 (IBM Corp., Armonk, N.Y., USA). Statistical analyses were carried out using logistic regression analysis. To estimate the risk of OFCs, the odds ratio (OR) was calculated at a confidence interval of 95% (95% CI). *P* values less than 0.05 were considered statistically significant.

## Results

A total of 323 cases and 400 controls were enrolled in this study. The mean ± SD of age was 31.4 ± 5.4 and 30.78 ± 5.05 years in the case and control mothers, respectively. Also, the mean ± SD of age was 32.60 ± 4.80 and 31.69 ± 4.76 years in the case and control fathers, respectively. Overall, 171 (53%) children in the case group and 204 (51%) children in the control group were female. Based on the results, 42 (13%) mothers of children with OFCs and 28 (7%) control mothers reported a history of abortion or miscarriage. Also, 119 (36%) mothers of children with OFCs and 93 (23%) control mothers reported a consanguineous marriage.

A review of mothers’ medical records showed that 36 (11%) mothers of children with OFCs and 32 (8%) control mothers suffered from at least one systemic disorder. Medication therapy during pregnancy was reported by 122 (38) case mothers and 124 (31%) control mothers. The results showed that 92 (29%) mothers of children with OFCs and 88 (22%) control mothers experienced a complication during pregnancy.

Based on the results, the correlation between the birth of a child with OFCs and child’s sex (OR = 1.081; CI 0.806–1.450), medication use during pregnancy (OR = 1.358; CI 0.997–1.850), and history of a systemic disorder (OR = 1.443; CI 0.874–2.380) was not significant (*P* > 0.05). However, the birth of a child with OFCs had a potential correlation with the maternal history of abortion/miscarriage (OR = 1.986; CI 1.201–3.283), consanguineous marriage (OR = 1.926; CI 1.393–2.662), and complications during pregnancy (OR = 1.412; CI 1.007–1.980) (*P* > 0.05).

Table 1 presents the results of bivariate and multivariate analyses, comparing the demographic and clinical characteristics of patients with OFCs and the controls. To improve the accuracy of our findings and prevent the effects of confounding factors, the adjusted ORs were also reported for the variables, in addition to the independent OR for each variable.

**Table 1:** The association between the risk factors and development of OF

Variables		Case (%)	Control (%)	Odds ratio (CI = 95%)	***P*** value	Adjusted OR^a^ (95% CI)	***P*** value
**Sex female (male, reference category)**		171 (53)	204 (51)	1.081 (0.806**–**1.450)	0.60354	1.007 (0.742**–**1.367)	0.964131
**History of abortion/miscarriage**		42 (13)	28 (7)	1.986 (1.201**–**3.283)	0.007475	1.339 (0.761**–**2.355)	0.311026
**Consanguineous marriage**		119 (36)	93 (23)	1.926 (1.393**–**2.662)	0.00735	1.854 (1.329**–**2.586)	0.0275
**Systemic diseases**		36 (11)	32 (8)	1.443 (0.874**–**2.380)	0.151417	1.307 (0.773**–**2.211)	0.318197
**Medication during pregnancy**		122 (38)	124 (31)	1.358 (0.997**–**1.850)	0.05254	1.339 (0.971**–**1.847)	0.074891
**Complications during pregnancy**		92 (29)	88 (22)	1.412 (1.007**–**1.980)	0.045517	1.293 (0.901**–**1.856)	0.162789
**Maternal age (years)**	**< 25**	43 (14)	48 (12)	1.483 (0.904**–**2.432)	0.118696	1.608 (0.952**–**2.717)	0.075617
	**26–30 (reference)**	87 (27)	136 (34)	1	**–**	1	-
	**30–35**	102 (31)	132 (33)	1.063 (0.939**–**1.204)	0.335031	1.062 (0.931**–**1.213)	0.369834
	**> 35**	91 (28)	84 (21)	1.151 (1.042**–**1.272)	0.005646	1.139 (1.027**–**1.264)	0.01355
**Paternal age (years)**	**< 25**	29 (9)	44 (11)	1.023 (0.584**–**1.792)	0.93643	1.092 (0.602**–**1.982)	0.771544
	**26–30 (reference)**	67 (21)	104 (26)	1	**–**	1	**–**
	**30–35**	122 (38)	144 (36)	1.098 (0.964**–**1.251)	0.158797	1.098 (0.958**–**1.260)	0.179591
	**> 35**	105 (32)	108 (27)	1.106 (0.998**–**1.225)	0.053716	1.105 (0.993**–**1.230)	0.067299
**Birth order**	**1st (reference)**	158 (49)	156 (39)	1	**–**	1	**–**
	**2nd**	83 (26)	140 (35)	0.760 (0.638**–**0.906)	0.002204	0.789 (0.656**–**0.948)	0.011335
	**3rd**	51 (16)	72 (18)	0.887 (0.771**–**1.021)	0.096105	0.914 (0.791**–**1.057)	0.225932
	**4th and higher**	31 (9)	32 (8)	0.997 (0.870**–**1.142)	0.963656	0.988 (0.856**–**1.142)	0.872614
**Total**		323(100)	400 (100)	**–**	**–**-	**–**	**–**

C

^a^OR is adjusted for all other factors in the table

## Discussion

The main purpose of this study was to evaluate the correlation between various risk factors and orofacial cleft disorder spectrum in newborns. As explained earlier, to improve the accuracy of available data, the correlation between the various risk factors and the risk of giving birth to a child with OFCs was evaluated in a multifactorial model. For this purpose, we reported the adjusted ORs, in addition to the crude ORs.

We may be able to computationally explain the incidence and prevalence of OFCs by examining the risk factors in each community [5]. This study was conducted at the Craniofacial and Cleft Research Center of Isfahan University of Medical Sciences, Isfahan, Iran. In this regard, Khazaei et al. [13], in a meta-analysis, reported that the cumulative incidence of OFCs in Iran was 1.0/1000 (0.77/1000 to 3.37/1000; 95% CI 0–1.5). Therefore, it can be concluded that the incidence of OFCs is lower in Iran, compared to the global average [10].

Our findings revealed a potential correlation between the history of abortion/miscarriage and the birth of a child with OFCs in the subsequent pregnancies (*P* < 0.05), which is consistent with the results of several studies [14–16]. Mbuyi-Musanzayi et al. [17] and Acuña-González et al. [18], in two independent studies, showed that the maternal history of miscarriage increased the risk of giving birth to a child with OFCs in the subsequent pregnancies; however, their findings were not statistically significant. According to our literature review, no study has yet assessed the etiology of the increased risk of OFCs in children of mothers with a history of miscarriage. It is theoretically possible that factors, such as consumption of anti-miscarriage medications, abnormalities and defects in the uterine wall, and increased maternal stress in the subsequent pregnancies, are related to the increased risk of OFCs in children of mothers with a history of miscarriage [19–22].

The results of the present study showed that female newborns were slightly more likely to develop cleft lip and cleft palate, compared to males, although this correlation was not significant (*P* > 0.05). The male/female ratio in our study was about 0.88, whereas this ratio in another study conducted in Iran was 2.3 [23]. Also, studies from other countries often suggest that males are more likely to be born with OFCs, compared to females [15, 17, 24]; overall, understanding the cause of this difference requires further investigations.

Moreover, medication use during pregnancy has been identified as a factor, which cannot potentially increase the risk of OFCs in children (*P* > 0.052). However, the independent analysis of medications used by mothers during pregnancy was beyond our capacity. Our investigations showed that the most common medications used by the mothers of children with OFCs during pregnancy included anti-stress medications, miscarriage prevention drugs, antibiotics, and some pain-relief medications, such as non-steroidal anti-inflammatory drugs. Nevertheless, the results of various studies [14–16, 25–28] regarding the increased risk of OFCs following medication therapy are inconsistent. We believe that it is not statistically reliable to report an absolute OR for all drug categories, and it is suggested to evaluate each drug category separately. The results of studies evaluating the effect of a particular drug on the increased risk of OFCs are probably more conclusive [29–32].

Pregnancy complications such as maternal cold or flu, preeclampsia, anemia, pregnancy hypertension, gestational diabetes, and intrauterine hypoxia can potentially increase the risk of OFCs in children (*P* < 0.05). Similarly, a study by Bui et al. [16] revealed that complications during pregnancy significantly increased the risk of OFCs in children. On the other hand, Acuña-González et al. reported that the effect of gestational diabetes and preeclampsia on the increased risk of OFCs was not significant (*P* > 0.05) [18].

According to the definition of systemic disorders, which are known to affect a number of organs and tissues or the body as a whole [33], we found that the effect of maternal systemic disorders on the risk of OFCs in children was not significant (*P* > 0.05). Similarly, the results of a study by Mirilas et al. [34] showed that maternal systemic disease could not significantly increase the risk of OFCs in children. On the other hand, Taghavi et al. [35] and Krapels et al. [36], in separate studies, reported that maternal systemic disease increased the risk of OFCs in children.

In the present study, the most remarkable factor, which potentially increased the risk of OFCs, was consanguineous marriage (*P* < 0.05). However, the results of studies by Mbuyi-Musanzayi et al. [17] and Corona-Rivera et al. [15] regarding the effect of consanguineous marriage on the risk of OFCs were not significant. It is estimated that 20% of the world’s population lives in communities, where there is an inclination toward consanguineous marriage, and 8.5% of children are the result of these marriages worldwide [37–39]. Overall, consanguineous marriage seems to be significantly influenced by cultural norms [40, 41]. The prevalence of consanguineous marriage in Iran is estimated at 38% (*α* = 0.0185), which is significantly higher than the global average [40, 42].

In the current study, the effects of maternal and paternal age were not significant for most categories. However, the results of a meta-analysis suggested that fathers and mothers above 40 years had 58% and 28% higher risks of having a child with OFC, respectively, compared to those aged 20–39 years. On the other hand, no association was observed between young maternal and paternal age and the occurrence of OFCs in another study [43].

Moreover, the results of our study regarding birth order showed that last children were more predisposed to OFCs, which is consistent with the findings of a meta-analysis [44].

Although a general pattern can be occasionally found among the risk factors for OFCs, our review of various studies indicated that the effects of these risk factors were not similar in different populations. Apart from differences in the sample size, study methodology, and statistical tests, the communities’ genetic diversities, as well as geographical, economic, and cultural factors, may affect the outcomes of studies conducted in different societies. For instance, the high prevalence of consanguineous marriage in relatively traditional developing countries significantly increases the risk of OFCs (e.g., Iran), compared to Western developed countries, where this type of marriage is not very common. The implicit conclusion that can be drawn from these findings is that studies conducted in different countries can only identify the factors, which may increase the risk of OFCs. However, to reach a reliable understanding of the factors increasing the risk of OFCs in a specific society, it is essential to conduct an exclusive study of risk factors in the target population.

In the present study, considering the wide range of OFC abnormalities with multiple underlying factors, it was not possible to examine all factors influencing OFCs due to the lack of facilities; this can be considered as the most important limitation of our study. Also, examination of different trimesters, a separate examination of different types of OFCs, paternal factors, smoking, consumption of alcohol, socioeconomic status of families, the status of maternal education, and maternal body mass index was among the crucial factors that could not be addressed in this study and require further investigation.

## Conclusion

Our results indicated a potential correlation between some variables such as consanguineous marriage or a maternal history of abortion/miscarriage and the birth of a child with OFCs in the subsequent pregnancies. Since the formation of OFC, lesions are not usually dependent on a single factor, and multiple cofactors may simultaneously contribute to such abnormalities, and a comprehensive, multidisciplinary care program is necessary to ensure a successful pregnancy period and birth of a healthy newborn. It is also necessary to conduct further studies in various populations in order to gain a comprehensive understanding of the multifactorial etiology of the OFCs disorder spectrum.

## Data Availability

The datasets generated and/or analyzed in the current study are not publicly available, but are available from the corresponding author on reasonable request.

## Abbreviations

OFCs: Orofacial clefts;
CL: Cleft lip
CP: Cleft palate
CLP: Cleft lip with cleft palate
OR: Odds ratio
CI: Confidence interval
